# A study on the impact of household head’s employment modes on the living standards of rural migrant workers’ families

**DOI:** 10.1371/journal.pone.0312518

**Published:** 2024-12-02

**Authors:** Qi Zhang, Yifei Yuan

**Affiliations:** School of Labor Economics, The Capital University of Economics and Business, Beijing, China; China University of Geosciences, CHINA

## Abstract

China has placed "mass entrepreneurship, mass innovation" as an important strategic deployment, and in 2020, China proposed further promoting the high-quality development of migrant workers returning to their hometowns for entrepreneurship. For migrant workers, the employment modes advocated by the above-mentioned policies correspond to starting a business in their own household registration location and returning to their hometowns for entrepreneurship. Therefore, this study aims to explore the impact of different employment modes of migrant workers on their family living standards. How much do they earn? How much do they spend on consumption? How much is left after deducting expenses? What is their subjective perception of living standards? And so on. By comparing and studying these aspects, it can provide a basis for future policy making and family decisions. Based on the China Household Income Project (CHIP) data, this paper compared the different impacts of employment modes of migrant worker household heads on subjective and objective family living standards through descriptive analysis, baseline regression analysis, heterogeneity analysis, and robustness tests. The following conclusions are drawn: the employment mode of the household head has significantly different impacts on the family living standards of migrant workers. Specifically, entrepreneurship increases household income by 25.71%, raises per capita consumption expenditure by 19.05%, and enhances the subjective perception of household living standards by 41.27% compared to being employed. Compared with working away from hometown, the income effect and consumption expenditure per capita effect of starting a business out of hometown are the highest, subjective perception effect is highest for local entrepreneurship. The gender, age, and whether they are household heads of the household head have different impacts on family living standards. At last, this paper discusses the choice of employment mode, household living standards, and the research value of household heads among rural migrant workers.

## 1. Introduction

According to the explanation of migrant workers in the China Rural Migrant Workers Monitoring and Survey Report, rural migrant workers refer to those who are still registered in rural areas and have been engaged in local non-agricultural industries or have been working outside for 6 months or more during the year. In China, since the reform and opening up, rural migrant workers have always occupied an important position in the labor market, and now the total number is about 280 million, accounting for one-third of the employed population, and they have become an important force driving China’s economic growth. Since the 21st century, due to the change of population age structure, rural migrant workers’ willingness to go out of town and regional economic development, some changes have occurred. While the total number of rural migrant workers keeps increasing, the growth rate starts to decline. The growth proportion of rural migrant workers going out of hometown decreases from 5.52% in 2010 to 1.48% in 2017, meanwhile, the proportion of local rural migrant workers increases. The proportion of local rural migrant workers in the total number of rural migrant workers reached 41.29% in 2021, an increase of 4.04% compared with ten years ago [1]. The number of entrepreneurs has increased, among which the number of migrant workers returning to their hometowns to start businesses has reached 4.5 million by 2016 [2].

The choice of employment status and region of migrant workers is not only about personal livelihood form, but also closely related to the living standard of their families. The head of the household, as the person in charge of the co-resident population, traditionally assumed the important responsibilities of the main source of economy and family decision-making. Although the perception of the head of the household has changed with the development of human society, the head of the household continues certain traditional functions especially in rural areas. Therefore, for rural migrant workers’ families, the choice of employment mode of the household head not only has an impact on their own life and development, but also has an important influence on the objective living conditions and subjective feelings of the family life.

This paper focuses on the rural migrant workers, takes the employment mode of the household head as the entry point and the family living standard as the research scope, uses the measurement method to quantify the degree and difference of the impact of the employment mode of the head of the household on the living standard of the household, and reveals the development pattern between the employment mode and the living standard of the household. The purpose is to gain a deep understanding of the basic issues of livelihood such as employment mode and living standard, so as to providing a basis for future policy development and household decision-making.

## 2. Literature review

Rural migrant workers is a unique group in China came out with the reform and opening up, and has made important contributions to social and economic development. After the implementation of the responsibility system of joint family production, peasants unleashed their great enthusiasm for labor and a large number of surplus rural laborers began to explore the practice of leaving the land or the countryside, creating wealth for the society, injecting vitality into urban and rural development, and making significant contributions to the modernization of the country. Some experts’ studies show that the average contribution of rural migrant workers to the GDP of non-farm industries was 16.37% during 1991–2010, and it showed a trend of increasing year by year, from 10.5% in 1991 to 19.4% in 2010 [3].

Farmers’ non-farm employment has an important impact on the perceptions and living standards of individuals and families, in addition to the national economic dimension. Some studies believe that that the imprinting effect brought by the non-agricultural employment experience, to a certain extent, changes the individual traits and risk preference types of them, enhances their confidence in production and operation [4]. In addition, the influence of urban consumption habits and consumption culture introduced by laborers moving to cities will enhance household consumption in food, clothing, and entertainment, and promote the rise of household living consumption level of rural residents [5], which in general promotes the happiness of rural residents [6].

Family living standard is a comprehensive reflection of the material and spiritual living conditions of family members, and is influenced by various factors such as individuals, families and the environment. The personal characteristics of core family members, such as gender, age, education level, health, and employment situation, are both a reflection of the individual’s ability to compete in society and an important aspect of the family’s living standard [7, 8], and some studies have shown that rural migrant workers with basic education, through their income from working and doing business to improve total family income level, which is conducive to the improvement of family resource allocation [9], and that an increase in the level of education significantly increases the happiness of rural migrant workers [10]; the characteristics of the family itself, such as the size of the family, how the dependency ratio, etc., also have an impact on family living standards [11, 12], some studies have shown that household size is significantly and positively related to income from agricultural production, income from non-farm production and other income, while per capita land ownership and stock of agricultural assets directly affect farm households’ income from agricultural production [13]; factors of macro environment, such as minimum wage level, social security system, regional economic development level, digital progress and application, etc. [14, 15] also have an impact on the living standard of rural migrant workers’ families.

Employment mode is a self-selection made under the constraint of supply and demand in the labor market, and different regions and employment categories will have an important impact on their personal income and thus on the living standard of their families. Employment is the basis of people’s livelihood, and it is the foundation for rural migrant workers to make a living and support their families. The choice of employment mode, whether to start a business or to work, and whether to work locally or abroad, implies differences in their labor profitability, living environment and lifestyle, which have different impacts on their families’ living standards. Some studies have shown that entrepreneurship among rural migrant workers has an effect of increasing income [16–19], but the degree of income increase is not agreed upon, ranging from as low as 6% to as high as 37.5% [16, 18, 20], while the increase in income from entrepreneurship and the expansion of the family social network promote residential consumption [21]. The impact on the household income and expenditure balance remains uncertain. self-employed rural migrant workers have higher satisfaction with their current life [20]; Studies indicate that migrant rural workers from other regions earn more than local rural migrant workers [22–24], but considering the cost of migration and living costs, the "increased income" brought by the field may not compensate for the cost of migration [22, 25]; the total household business income of farming households increases significantly due to outworking, and it mainly comes from the increase of non-farm production income [11]. But there are some studies have found that the average contribution of labor who work out of hometown and local labor (including work and farming) to rural household income is 23.8% and 22.5% respectively, with a small gap, indicating that working away from home contributes slightly more to income but not significantly [26].

The head of the household is of great significance in studies on households and has an important impact on household financial assets, income and expenditure. In relevant studies, the head of household is the head of the co-resident members and the manager of household income and expenditure [27], and the head of household is more often male in the traditional perception, but with the changing times, the gender of the head of household is no longer bound to be as the past popular perception, and the number of female heads of household is increasing. Some studies have shown that the age of the household head significantly affects the household’s household rent and purchase decisions [28], female-headed households hold less subsistence capital and have higher poverty vulnerability compared to male-headed households [12]. An increase in the education level of the household head has a positive impact on the participation and percentage of risky financial assets held [29]. Household head’s education Increasing education level significantly reduces household savings rate by reducing income uncertainty [30].

**In summary**, the existing literature, fully affirms that rural migrant workers are an important force in China’s social and economic development, and that rural migrant workers, with their hard-working spirit, whether locally or abroad, or starting or working in business, have been baptized by the market economy, transformed their attitudes and improved their family living standards. **However, there are also the following limitations: first,** regarding the research on household head, the existing literature acknowledges the influence of age, gender and education of household head on the household, but there are very few studies that start from the employment mode of the household head and explore its influence on the living standard of the household, while in fact the influence of the household head on the household economy is more mainly reflected by the income obtained through the employment mode. **Secondly,** the research on family living standard is mostly focused on objective indicators of income and consumption, and ignores the subjective feeling of living standard, but in fact, subjective living feeling is also an important measure of family living standard. The combination of objective and subjective indicators of living standards allows for a more comprehensive picture of the household’s living standard situation. **Thirdly,** the research on employment mode is mostly focused on rural migrant workers working outside the home or starting their own business, and its impact on personal income, but there are fewer articles studying its impact on families from the perspective of individual employment choices, and there is also a lack of systematic consideration of the organic combination of employment area and employment mode.

Therefore, this paper uses the employment mode choice of the household head as the independent variable to explore its impact on the household living standard as well as heterogeneity. In this paper, employment mode is not only classified into entrepreneurship and labor according to employment status, but also subdivided into four employment mode choices by combining employment status and employment region as subdivision criteria inspired by China’s advocacy of migrant workers returning to their hometowns to start their own businesses, and household living standard is selected from both subjective and objective aspects with a total of three indicators: household disposable income, household economic balance, and self-assessed household living standard. **An attempt is made to answer the following questions:** First, what is the effect of the household head’s choice of employment mode on the household’s standard of living? Are there household income effects, household economic balance effects, and subjective perception effects? Second, are there any gender and age heterogeneity in the effects of household head’s employment mode choice on household living standards? Are there any differences between household heads and non-household heads?

**Possible contributions of this paper:** first, from the micro household perspective, the employment mode choice of household heads is linked to household living standard, which enriches the perspective and content of this study and to some extent makes up for the deficiencies of existing studies. Second, objective and subjective indicators are integrated to measure household living standard, and four indicators are selected, namely, household disposable income, consumption expenditure per capita, household economic balance, and self-assessed household living condition, which more comprehensively. Third, we systematically consider the possible employment modes of rural migrant workers, combine the employment status with the employment area, and refine the impact of various employment modes of the household head on the household living standard.

## 3. Data and methods

### 3.1. Data sources

The data used in this paper is from the China Household Income Survey (CHIP), which is conducted and executed by the China Institute of Income Distribution, Beijing Normal University, in cooperation with the National Bureau of Statistics. The survey respondents come from the National Bureau of Statistics’ annual urban-rural integrated regular household survey large sample pool, which covers 160,000 households in all 31 provinces (municipalities and autonomous regions), and the CHIP project team draws the respondents of the CHIP according to the systematic sampling method by stratifying according to the East, Central and West. The China Household Income Survey (CHIP) has been conducted six household surveys successively in 1989, 1996, 2003, 2008, 2014 and 2019. They collected income and expenditure information, as well as other household and personal information in 1988, 1995, 2002, 2007, 2013 and 2018, respectively, and were numbered CHIP1988, CHIP1995, CHIP2002, CHIP2007, CHIP2013 and CHIP2018.Sampling was conducted according to the systematic sampling methodology, with scientific methodology the total number of samples is rich and of high quality. The data content of the database includes basic information and employment information at the individual household level, as well as basic information, major income and expenditure information and some thematic issues at the household level. The database covers the research objects and related indicators needed in this paper, and meets the basic requirements of data sources for empirical research. In this paper, the latest 2018 survey database is selected as the benchmark regression sample pool.

Considering the research object of this paper, this thesis selects the sample of agricultural households, engaged in non-farm employment, aged 16–64 years old and with the status of head of household from the full sample, and finally obtains 5,407 valid samples of rural migrant workers’ heads of household after considering the validity of relevant factors and deleting missing values, outliers, etc.

### 3.2. Variable selection and data description

The outcome variables. The outcome variable of this paper is household living standard, including objective living standard and subjective living perception. This paper chose household disposable income, Consumption Expenditure per capita, household economic balance and self-assessed household living standard as specific indicators. Among them, the "household disposable income" is selected from the "Disposable income (household)" in the questionnaire, “Consumption Expenditure per capita” is the result of dividing total living consumption expenditures by total household size, the "household economic balance" is the result of deducting the "Disposable income (household)" from the "consumption expenditure (household)" in the questionnaire, the question “Compared to other households in your region, do you consider your household’s living standards to be?” was chosen to measure the level of subjective household living based on the design of the CHIP questionnaire, "self-assessed household living standard" is the different responds of it and the response options were “Substantially below average”, “Somewhat below average”, “About average”, “Somewhat above average” and “Substantially above average”, which were assigned a value of 1, 2, 3, 4 and 5, respectively.

Explanatory variable. The explanatory variable in this paper is employment mode. The employment mode can be divided into “employer” and “employee” according to employment status. Among them, rural migrant workers whose "employment status" in the questionnaire is “employer” or “self-employed” are classified as entrepreneurship, while those whose "employment status" is "employee" are counted as laborers. Rural migrant workers whose "current place of residence" and "most important place of work" are both in their own township and who have lived there for more than six months during the year are counted as local rural migrant workers. Combining both employment status and region, the employment mode can be divided into four types of employment: "entrepreneurship out of hometown", "local entrepreneurship", "being hired out of hometown" and "being hired locally".

Other control variables. According to the factors influencing the living standard of households summarized in the literature, the control variables in this paper are divided into three categories: personal characteristics of household head, household characteristics, and environmental factors, among which, personal characteristics of household head include: gender, age, marital status, education level, and health status of household head; household characteristics include: household size, population dependency ratio, and arable land area; environmental factors include: regional GDP per capita index. Only the regional economic development indicator is selected because this paper uses cross-sectional data, the macro institutional environment is relatively consistent. The assignment of all variables is shown in Table 1, and the descriptive statistics of variables are shown in Table 2.

**Table 1:** Description of variable classification and assignment.

	Category Breakdown	Variable	Situation description
Dependent variables	Objective household living standard	Household disposable income	Continuous variable, total disposable income in 2018
		Consumption Expenditure per capita	Continuous variable, consumption expenditure per capita = consumption expenses/household size
		Household economic balance	Continuous variable, household economic balance = disposable income—consumption expenses
	Subjective household living standard	Self-assessed household living	Level ordinal variable, Compared to other households in your region, do you consider your household’s living standards to be? 1 = “Substantially below average”, 2 = “Somewhat below average”, 3 = “About average”, 4 = “Somewhat above average”, 5 = “Substantially above average”
Explanatory variables	Employment mode	Whether to start a business;	1 = Starting a business, 0 = Working
	Combination of employment status and geographical area of employment	Local entrepreneurship;	Whether to start a business locally, 1 = yes, 0 = no
		Entrepreneurship out of hometown	Whether start a business out of hometown, 1 = yes, 0 = no
		Being hired locally;	Whether being hired locally, 1 = Yes, 0 = No
		Being hired out of hometown	Whether being hire out if hometown, 1 = yes, 0 = no
Control variables	Characteristics of household head	Gender	Categorical variable, 1 = male, 0 = female
		Age	Continuous variable, unit: years
		Marital status	Categorical variable, 1 = married (first marriage, remarriage), 0 = other
		Educational attainment	Ordinal variable, 1 = never attended school, 2 = elementary school, 3 = junior high school, 4 = high school, 5 = vocational/technical school, 6 = junior college, 7 = junior college, 8 = undergraduate college, 9 = graduate
		Health status	Ordinal variables, your current health condition 1 = Very poor, 2 = Poor, 3 = Average, 4 = Good, 5 = Excellent
	Household characteristics	Household size	Discrete variable, unit: person
		Population dependency ratio	Continuous variable, population dependency ratio = non-working population/working population
		Arable land area	Continuous variable, unit: mu
	Regional characteristics	Regional GDP per capita	GDP per capita by region in 2018

Note: Data on regional GDP per capita are from *China Statistical Yearbook 2019 [31]*, compiled by the National Bureau of Statistics of the People’s Republic of China

**Table 2:** Statistics of objectivity facts of each variable.

Variable	Mean	Std. Dev.	Min	Max	Obs
Household disposable income	63602.35	51695.68	5389.09	362436.70	5,407
Household disposable income (in logarithm)	10.80	0.74	8.59	12.80	5,407
Consumption Expenditure per capita	16001.28	17220.84	1434.39	302584	5,407
Consumption Expenditure per capita(in logarithm)	9.40	0.70	7.27	12.62	5,407
Household economic balance	15752.23	40474.06	-98648.97	189956.40	5,407
Household economic balance (in logarithm)	11.49	1.16	0	12.57	5,407
Self-assessed household living standard	3.02	0.75	1	5	5,407
Whether to start a business	0.22	0.41	0	1	5,407
Starting a business locally	0.13	0.33	0	1	5,407
Starting a business abroad	0.09	0.29	0	1	5,407
Being hired locally	0.37	0.48	0	1	5,407
Being hired abroad	0.41	0.49	0	1	5,407
Gender	0.93	0.26	0	1	5,407
Age	43.21	9.88	17	64	5,407
Marital status	0.94	0.24	0	1	5,407
Educational level	3.16	1.25	1	9	5,407
Health	4.08	0.86	1	5	5,407
Family size	3.51	1.32	1	10	5,407
Population dependency ratio	0.34	0.43	0	4	5,407
Arable land area (in logarithm)	1.51	0.91	0	7.71	5,407
Regional GDP per capita (in logarithm)	10.99	0.34	10.35	11.85	5,407

### 3.3. Model construction

First, the corresponding models are selected according to the different types of dependent variables. Since household disposable income and household disposable balance are continuous variables, the ols model is selected for parameter estimation, and the model is constructed as follows.


$$Y_{i}=\alpha +\beta_{1}{work}_{i}+\delta X_{i}+\varepsilon_{i}$$ (1)

Among them, ***Y***_***i***_ is a series of indicators for household living standard, including household disposable income, consumption expenditure per capita and household economic balance, then ***α*** is the intercept term, ***work***_***i***_ represents employment modes, covariate matrix ***X***_***i***_ is the control variables, including personal characteristics of household head, family characteristics, environmental factors, etc., then ***ε***_***i***_ is the random disturbance term.

Meanwhile, because the self-assessed household living standard is an ordered multicategorical variable, the ologit model is selected for parameter estimation, and the model is constructed as follows in this paper, drawing on the research method of Lian Yujun [32].


$$Y_{i}=F(\beta_{2}{work}_{i}+\delta_{2}X_{i}+\varepsilon_{i})$$ (2)

In Eq (2), ***Y***_***i***_ is the explanatory variable, representing the self-assessed household living standard, ***work***_***i***_ is the employment mode, and the covariate matrix ***X***_***i***_ is the set of control variables, including the personal characteristics of the household head, family characteristics, environmental factors, etc. See Table 1 for details, then ***ε***_***i***_ is the random disturbance term.F(•) is a nonlinear function, and the four consecutive segments into which it falls are noted as F(Y_i_*),then we have.

$$F(Y_{i}*)=\{\begin{array}{ll} 1, & Y_{i}*\leq r_{1}\\ 2, & r_{1}\leq Y_{i}*\leq r_{2}\\ 3, & r_{2}\leq Y_{i}*\leq r_{3}\\ 4, & r_{3}\leq Y_{i}*\leq r_{4}\\ 5, & r_{4}\leq Y_{i}* \end{array}$$ (3)

where are the parameters to be estimated, called "cut points". * is a continuous variable, which is not observable behind Y. It is called a latent variable and satisfies.


$$Y_{i}=\beta_{2}{work}_{i}+\delta_{2}X_{i}+\varepsilon_{i}$$ (4)

Second, to avoid possible self-selection bias between employment mode and household living standards, we use propensity score matching (*Propensity Score Matching*, *PSM* for short) to measure the net utility of the effect of different employment modes of the household head on household living standards. Taking whether the household head starts a business or not as an example, the specific approach is to divide the sample into a treatment group (*y*_1*i*_) for starting a business and a control group (*y*_0*i*_) for working, calculate the propensity score for each individual in the treatment group based on a series of characteristics of the control variables, find a match in the control group, and then obtain two balanced samples, and then calculate the average treatment effect (*ATT*) based on the matched treatment and control groups, which can avoid This can avoid the influence and interference brought by sample bias and estimate the effect between the two more accurately. The general expression is as follows

$$ATT=E(y_{1i}-y_{0i}|D_{i}=1)$$ (5)

where ***D***_***i***_ is the treatment variable, and a value of 1 indicates that the individual i is in the treatment group, i.e., starting a business; a value of 0 indicates that the individual is in the control group, i.e., working. *y*_1*i*_, *y*_0*i*_ denote the estimated results for the treatment and control groups, respectively. ***ATT*** denotes the average treatment effect of the effect of different employment modes of the household head on the household living standard.

## 4. Empirical analysis

### 4.1. Descriptive statistical analysis

In order to understand the relationship between employment mode of household head and household living standard, this paper lists different employment modes of household head, household living standard and the relationship between them in Table 3, from which it can be found that: first, from the employment mode, the proportion of household head starting a business accounts for 21.82%, which is much lower than the proportion of 78.18% for working, combined with the employment region, the proportion of local entrepreneurs is higher than the proportion of entrepreneurs out of hometown, and the proportion of employees out of hometown is higher than that of local employees. Employment mode is the result of market supply and demand, and the above data shows that the survey respondents are more likely to find labor opportunities out of homelands and more likely to find entrepreneurial opportunities in local areas. Second, in terms of family living standard, as the objective indicators shows, the average value of household disposable income is 63,602.35 yuan, the average consumption expenditure per capita is 16001.28 yuan, the average value of household economic balance is 15,752.23 yuan, and as the subjective indicator shows that the self-assessed family living standard is 3.02. Third, In terms of the relationship between the two, entrepreneurship outperforms work in all variables of household living standards, indicating that entrepreneurship brings better benefits and rewards. This is understandable because entrepreneurship is complex and challenging, requiring more energy and input, and the success in entrepreneurship also means higher payback and a sense of accomplishment, which naturally has a better impact on family living standards than being employed. From the objective indicators of family living standard, entrepreneurship out of hometown is higher than local entrepreneurship and doing work out of hometown is better than doing work in hometown, which shows that the main motivation for rural migrant workers to leave their hometown is economic consideration; while the subjective indicators of family living standard overturn the conclusion of objective indicators and show that in the hometown is better than other places. This may be related to the fact that local employment is easier to enjoy the warmth of family reunion and mutual care, which may be one of the reasons for the increase of rural migrant workers returning to their hometown in recent years.

**Table 3:** Mean statistics of employment mode of household head and family living standard.

	Percentage (%)	Household disposable income (yuan)	Consumption Expenditure per capita(yuan)	Household economic balance (yuan)	Self-assessed household living standard
Entrepreneurship (N = 1180)	21.82	83252.72	18757.34	24690.77	3.16
Working (N = 4227)	78.18	58116.8	15231.91	13256.97	2.98
Local(N = 2700)	49.94	58056.74	13503.41	14821.55	3.06
out-of-town(N = 2700)	50.06	69133.63	18492.7	16680.51	2.98
Full sample(N = 5407)	100	63602.35	16001.28	15752.23	3.02
Local entrepreneurship(N = 685)	12.67	69099.06	14484.12	19879.52	3.19
Nonlocal entrepreneurship(N = 495)	9.15	102839.1	24670.79	31348.75	3.11
Local workers(N = 2015)	37.27	54302.9	13170.02	13102.09	3.02
Out-of-hometown workers (N = 2212)	40.91	61591.04	17110.17	13398.06	2.95

### 4.2. Baseline regression analysis

The impact of the employment mode of the household head on the living standard of rural migrant workers’ households is analyzed according to Equations. Since the outcome variables are different types of variables, different measurement methods are used. Since household disposable income, consumption expenditure per capita and household economic balance are continuous variables, the analysis is conducted using ols basic regression method, while self-assessed household living standard is ordered variable, so the analysis is conducted using ologit ordered classification model. At the same time, the evaluation of subjective family living standard is to some extent influenced by the objective economic conditions of the family, so the disposable income of the family is also a control variable for the self-assessed family living standard. The explanatory variable, employment mode, we analyzed from two perspectives, one is to classify the employment mode into entrepreneurship and work, and the other is to assemble the employment mode with the employment area into four categories: local entrepreneurship, foreign entrepreneurship, local labor and foreign labor. The specific regression results are shown in Table 4.

**Table 4:** Impact of different employment modes of household heads on rural migrant workers’ family living standards.

	Household disposable income	Consumption Expenditure per capita	Household economic balance	Self-assessed household living standard	Household disposable income	Consumption Expenditure per capita	Household economic balance	Self-assessed household living standard
Entrepreneurship vs. Work	0.2571***	0.1905***	-0.0346	0.4127***				
	(0.0248)	(0.0197)	(0.0481)	(0.0675)				
Local entrepreneurship vs. Working out of hometown					0.1321***	0.0620**	0.0050	0.5645***
					(0.0313)	(0.0251)	(0.0518)	(0.0860)
Nonlocal Entrepreneurship vs. Working out of hometown					0.4022***	0.3047***	-0.1091	0.3423***
					(0.0396)	(0.0311)	(0.0916)	(0.1071)
Working in hometown vs. Working out of hometown					-0.0278	-0.0603***	-0.0170	0.1370**
					(0.0208)	(0.0176)	(0.0328)	(0.0616)
Gender	-0.2651***	-0.1958***	0.0503	0.3592***	-0.2610***	-0.1933***	0.0477	0.3605***
	(0.0347)	(0.0312)	(0.0866)	(0.1169)	(0.0340)	(0.0307)	(0.0868)	(0.1167)
Age	-0.0079***	-0.0135***	0.0055***	0.0224***	-0.0068***	-0.0121***	0.0054***	0.0202***
	(0.0011)	(0.0009)	(0.0020)	(0.0034)	(0.0011)	(0.0010)	(0.0021)	(0.0034)
Marital status	0.2815***	0.1464***	-0.0629	0.3718***	0.2768***	0.1420***	-0.0610	0.3749***
	(0.0380)	(0.0340)	(0.0494)	(0.1274)	(0.0377)	(0.0336)	(0.0494)	(0.1274)
Educational attainment	0.1307***	0.1225***	-0.0004	0.0329	0.1297***	0.1217***	0.0001	0.0325
	(0.0082)	(0.0070)	(0.0165)	(0.0267)	(0.0081)	(0.0069)	(0.0165)	(0.0266)
Health	0.0533***	0.0054	0.0220	0.4040***	0.0509***	0.0029	0.0228	0.4075***
	(0.0114)	(0.0095)	(0.0214)	(0.0338)	(0.0113)	(0.0094)	(0.0214)	(0.0338)
Household size	0.0744***	-0.1972***	-0.0047	0.0759***	0.0768***	-0.1952***	-0.0058	0.0735***
	(0.0081)	(0.0071)	(0.0124)	(0.0241)	(0.0081)	(0.0071)	(0.0123)	(0.0241)
Population dependency ratio	-0.1254***	-0.0768***	-0.0582	-0.1985**	-0.1287***	-0.0786***	-0.0559	-0.1970**
	(0.0239)	(0.0205)	(0.0419)	(0.0785)	(0.0238)	(0.0203)	(0.0415)	(0.0785)
Arable land area	0.0112	-0.0327***	0.0230	-0.0175	0.0081	-0.0354***	0.0243	-0.0147
	(0.0108)	(0.0084)	(0.0179)	(0.0284)	(0.0108)	(0.0084)	(0.0177)	(0.0284)
Regional GDP per capita	0.4412***	0.2429***	0.1084**	-0.0129	0.4415***	0.2439***	0.1088**	-0.0197
	(0.0268)	(0.0238)	(0.0501)	(0.0850)	(0.0267)	(0.0236)	(0.0502)	(0.0854)
Household disposable income				0.2572***				0.2649***
				(0.0416)				(0.0419)
_cons	5.3467***	7.6727***	9.9982***		5.3197***	7.6401***	10.0027***	
	(0.3017)	(0.2632)	(0.5434)		(0.3008)	(0.2615)	(0.5458)	
R2/	0.173	0.353	0.006		0.180	0.361	0.006	
adj. R2/	0.1720	0.3520	0.0038		0.1787	0.3592	0.0040	
Prob > chi2				0.0000				0.0000
Log pseudolikelihood				-5861.1482				-5857.1684
Pseudo R2				0.0299				0.0306
N	5407	5407	5407	5407	5407	5407	5407	5407

Note: Robust standard errors are in parentheses. *, **, *** denote significant at the 10%, 5%, and 1% levels, respectively. Same below.

From Table 4, it can be seen that.

**First,** in terms of objective indicators of household living standards, the effects of the household head’s employment mode on household disposable income and consumption expenditure per capita are partially significant, but none of the effects on household economic balance are significant.

In terms of household disposable income indicators, when controlling for other variables, entrepreneurship increases household disposable income by 25.71% compared to being employed, entrepreneurship out of hometown raises income by 40.22% over working out of hometown, and local entrepreneurship raises income by 13.21% over being hired out of hometown, all of which are significant at the 1% level, and only local work has a negative but insignificant effect on household disposable income compared to working out of hometown. This regression result is basically consistent with the findings of the descriptive analysis and is largely consistent with the findings of Huang Zhiling [19], Zhu Zhisheng [20], Ning Guangjie [22], Han Junhui [23] and others in terms of the direction of impact despite the different levels of impact. The result shows that the income effect of entrepreneurship is significant and the income effect in the field is greater than the income effect in hometown. However, the negative but insignificant effect of local workers compared to foreign workers on household disposable income is not consistent with the perception that out-of-hometown workers earn more than local workers, but this finding is similar to the conclusion obtained by Zhen Xiaopeng et al. [26] that the difference between the contribution of being employed out of hometown and being employed locally to household income is not significant.

In terms of consumption expenditure per capita, when controlling for other variables, entrepreneurship increases consumption expenditure per capita by 19.05% compared to being employed, entrepreneurship out of hometown consumption expenditure per capita by 6.2% over working out of hometown, and local entrepreneurship raises consumption per capita by 30.47% over being hired out of hometown and being employed locally reduces consumption by 6.03% compared to working out of hometown, all of which are significant.

Looking at the indicator, household economic balance, the effect of the employment mode of the household head on it is not significant when controlling for other variables, because economic balance is the difference between income and consumption. Comparing the empirical results for income and consumption, it can be seen that consumption expenditure per capita is almost just following the rise in income, which may be the main reason for the insignificant impact of employment patterns on household economic balance.

**Second,** in terms of subjective indicators of household standard of living, the employment mode of the household head has a significant positive effect on the perceived standard of living of the own household compared to other households in their region. Specifically, when controlling other variables, entrepreneurship improves self-rated household living standards by 41.27% over being employed, local entrepreneurship improves self-rated household living standards by 56.45% over being employed out of hometown, starting business out of hometown improves self-rated household living standards by 34.23% over being employed out of hometown, and being employed locally improves self-rated household living standards by 13.70% over being employed out of hometown. This regression result is largely consistent with the findings of the descriptive analysis, which shows that entrepreneurship is significantly more effective in improving subjective living standards, with better perceived family living standards in hometown than out of hometown. This finding is similar to other scholars’ studies, where previous studies found that entrepreneurship can positively affect happiness through income effects [10], while working outside the home negatively affects the happiness of rural migrant workers due to social network limitations and insufficient social participation [33], which may also be the reason why entrepreneurship has better impact on self-rated family living standard than working and local employment than foreign employment.

**Third,** in terms of control variables, control variables basically have significant effects on household disposable income, consumption expenditure per capita and self-rated household living standards, while almost all indicators have insignificant effects on household economic balance. Specifically, having a spouse, being healthier, and having a higher level of education have positive and significant effects on household disposable income, while as the population dependency ratio increases, the household standard of living subsequently decreases, consistent with common sense. Higher regional GDP per capita and better economic development are conducive to the objective living standard indicators of rural migrant workers’ households but have no significant effect on rural migrant workers’ self-assessed living standards.

### 4.3. Endogeneity test

In this paper, the instrumental variables method and propensity score matching are used to overcome endogeneity.

#### 4.3.1. IV

There may be the problem of omission of key variables affecting the accuracy of the estimation, so the instrumental variable method was adopted, using the ratio of the number of people engaged in each mode of employment to total number in each province as an exogenous instrumental variable for each corresponding employment mode. For example, the instrumental variable for the dependent variable entrepreneurship is the ratio of provincial entrepreneurship to total employment. As another example, the instrumental variable corresponding to the independent variable local entrepreneurship is the ratio of the number of people engaged in local entrepreneurship at the provincial level to the total number of people employed, and so on.

The use of provincial employment style ratios as an instrumental variable is appropriate because this is associated with a certain employment style behavior of the individual. At the same time, it is unlikely to directly affect the micro-level income, consumption, balance of income and expenditure, and subjectively perceived standard of living at the micro level of the household, fulfilling the exogeneity requirement for instrumental variables [34].

The results are shown in Table 5.

**Table 5:** Impact of the employment mode of the head of household on the standard of living of migrant workers’ families obtained using the IV methodology.

	Household disposable income	Consumption Expenditure per capita	Household economic balance	Self-assessed household living standard	Household disposable income	Consumption Expenditure per capita	Household economic balance	Self-assessed household living standard
Entrepreneurship vs. Work	0.2602***	0.2043***	-0.0413	0.1501***				
	(0.0248)	(0.0202)	(0.0490)	(0.0249)				
Local entrepreneurship vs. Working out of hometown					0.0965***	0.0505*	-0.0500	0.2392***
					(0.0329)	(0.0267)	(0.0547)	(0.0338)
Nonlocal Entrepreneurship vs. Working out of hometown					0.3793***	0.3161***	-0.1651*	0.1520***
					(0.0405)	(0.0326)	(0.1001)	(0.0406)
Working in hometown vs. Working out of hometown					-0.1020***	-0.0932***	-0.1025**	0.1102***
					(0.0286)	(0.0230)	(0.0472)	(0.0315)
Gender	-0.2649***	-0.1952***	0.0500	0.1293***	-0.2629***	-0.1933***	0.0442	0.1322***
	(0.0347)	(0.0311)	(0.0865)	(0.0432)	(0.0337)	(0.0306)	(0.0866)	(0.0431)
Age	-0.0079***	-0.0135***	0.0054***	0.0078***	-0.0058***	-0.0116***	0.0064***	0.0061***
	(0.0011)	(0.0009)	(0.0020)	(0.0013)	(0.0011)	(0.0010)	(0.0021)	(0.0013)
Marital status	0.2813***	0.1455***	-0.0625	0.1218***	0.2760***	0.1406***	-0.0602	0.1226***
	(0.0380)	(0.0340)	(0.0493)	(0.0470)	(0.0376)	(0.0334)	(0.0494)	(0.0469)
Educational attainment	0.1307***	0.1226***	-0.0004	0.0112	0.1298***	0.1217***	-0.0000	0.0110
	(0.0082)	(0.0070)	(0.0165)	(0.0095)	(0.0081)	(0.0069)	(0.0165)	(0.0095)
Health	0.0532***	0.0051	0.0222	0.1480***	0.0500***	0.0021	0.0224	0.1495***
	(0.0114)	(0.0095)	(0.0214)	(0.0126)	(0.0113)	(0.0094)	(0.0214)	(0.0126)
Household size	0.0744***	-0.1973***	-0.0046	0.0330***	0.0766***	-0.1952***	-0.0060	0.0324***
	(0.0081)	(0.0071)	(0.0124)	(0.0090)	(0.0081)	(0.0071)	(0.0124)	(0.0090)
Population dependency ratio	-0.1254***	-0.0770***	-0.0581	-0.0780***	-0.1265***	-0.0781***	-0.0528	-0.0799***
	(0.0239)	(0.0204)	(0.0419)	(0.0298)	(0.0238)	(0.0203)	(0.0413)	(0.0297)
Arable land area	0.0112	-0.0325***	0.0229	-0.0041	0.0080	-0.0355***	0.0241	-0.0032
	(0.0108)	(0.0084)	(0.0178)	(0.0106)	(0.0108)	(0.0085)	(0.0177)	(0.0107)
Regional GDP per capita	0.4413***	0.2433***	0.1082**	0.0045	0.4431***	0.2449***	0.1101**	0.0010
	(0.0268)	(0.0238)	(0.0500)	(0.0313)	(0.0267)	(0.0236)	(0.0500)	(0.0314)
Household disposable income				0.0904***				0.0937***
				(0.0151)				(0.0152)
_cons	5.3451***	7.6658***	10.0015***	0.6717*	5.2993***	7.6240***	9.9905***	0.6853*
	(0.3013)	(0.2630)	(0.5429)	(0.3492)	(0.3008)	(0.2613)	(0.5442)	(0.3505)
Anderson canon. corr. LM statistic	1849.513***	1849.513***	1849.513***	1814.837***	2497.868***	2497.868***	2501.799***	2501.799***
Cragg-Donald Wald F statistic	1.4e+05	1.4e+05	1.4e+05	1.4e+05	2497.868	2573.616	2573.616	2501.799
R2	0.173	0.353	0.006	0.063	0.179	0.360	0.005	0.064
adj. R2	0.1720	0.3520	0.0038	0.0613	0.1769	0.3587	0.0030	0.0614
N	5407	5407	5407	5407	5407	5407	5407	5407

Note: Robust standard errors are in parentheses. *, **, *** denote significant at the 10%, 5%, and 1% levels, respectively. Same below.

As can be seen in Table 5, the results of the benchmark regression are more robust, with all model instrumental variable tests passing the non-identifiable test (p<0.01), the weak instrumental variable test (Cragg-Donald Wald F-statistic is significantly greater than 10 and the smallest eigenvalue statistic is greater than the critical value of 5%), which further proves that the selection of instrumental variables in this chapter is justified and that the model is valid.

#### 4.3.2. PSM

The employment mode of migrant workers is the result of their independent choice, which may be affected by the human capital of migrant workers and so on, while at the same time their human capital will have an impact on the standard of living of the household, thus generating the problem of self-selection of the sample, the propensity score matching method is used to overcome the problem so as to obtain a more robust regression result.

The Propensity Score Matching (PSM) method constructs a counterfactual framework and re-matches the sample for analysis to correct for selectivity bias, and the net effect of the impact of household head’s employment style on household standard of living obtained by using the kernel matching method is shown in Table 6.

**Table 6:** Net effect of the impact of the employment mode of the household head on the living standard of rural migrant workers’ households obtained using the PSM method.

PSM	Household disposable income	Consumption Expenditure per capita	Household economic balance	Self-assessed household living standard
Entrepreneurship vs. Work	0.2580***(0.0264)	0.1887***(0.0236)	-0.0420(0.0491)	0.1636***(0.0248)
Local entrepreneurship vs. Working out of hometown	0.1436***(0.0339)	0.0674**(0.0292)	0.0127(0.0530)	0.2060***(0.0327)
Nonlocal Entrepreneurship vs. Working out of hometown	0.4161***(0.0413)	0.3093***(0.0385)	-0.1089(0.0930)	0.1480***(0.0395)
Working in hometown vs. Working out of hometown	-0.0354(0.0241)	-0.0516**(0.0234)	-0.0008(0.0344)	0.0413(0.0254)

Note: *, **, *** indicate significant at the 10%, 5%, and 1% levels, respectively. Robust standard errors are in parentheses.

From Table 6, it is clear that the results of the baseline regression are broadly robust. After overcoming the self-selection bias, the results of the propensity score matching method are basically the same as those of the basic regression, with slight differences in the magnitudes of the coefficients. It is worth noting that there is one significance differ from the basic regression, the positive household income effect of local employees relative to foreign employees changes from significant to insignificant. The possible reason is that the better perception of living standards associated with local being hired, compared to out-of-town being hired, may be partially offset by similar income levels and lower consumption expenditures.

### 4.4. Expanded analysis

In order to better grasp the family effect situation of employment mode and understand the impact of employment mode on the living standard of migrant workers’ families with different family living standards, this paper chooses applicable methods to deal with the three selected types of indicators of family living standards differently, respectively, and carries out quantile regression for the continuous dependent variable, and marginal effect analysis of olit model for ordered categorical variables, and obtains the results as shown in Tables 7 and 8.

**Table 7:** Results of quantile regression estimating the impact of employment mode of migrant workers’ head of household on different household living standards.

Household disposable income	Ols	Q = 0.10	Q = 0.25	Q = 0.50	Q = 0.75	Q = 0.90
Local entrepreneurship vs. Working out of hometown	0.1321***	0.1322**	0.1101**	0.0835**	0.0732*	0.1745***
	(0.0298)	(0.0662)	(0.0429)	(0.0333)	(0.0383)	(0.0558)
Nonlocal Entrepreneurship vs. Working out of hometown	0.4022***	0.1650**	0.3186***	0.3866***	0.5000***	0.5509***
	(0.0336)	(0.0747)	(0.0483)	(0.0375)	(0.0431)	(0.0629)
Working in hometown vs. Working out of hometown	-0.0278	0.0763	0.0308	-0.0284	-0.0920***	-0.1099***
	(0.0217)	(0.0481)	(0.0311)	(0.0242)	(0.0278)	(0.0405)
control variables	Y	Y	Y	Y	Y	Y
Consumption Expenditure per capita	Ols	Q = 0.10	Q = 0.25	Q = 0.50	Q = 0.75	Q = 0.90
Local entrepreneurship vs. Working out of hometown	0.0620**	0.0711*	0.0427	0.0608**	0.0736**	0.1298***
	(0.0247)	(0.0382)	(0.0331)	(0.0277)	(0.0344)	(0.0476)
Nonlocal Entrepreneurship vs. Working out of hometown	0.3047***	0.2811***	0.2396***	0.2463***	0.3781***	0.4200***
	(0.0279)	(0.0431)	(0.0373)	(0.0312)	(0.0388)	(0.0537)
Working in hometown vs. Working out of hometown	-0.0603***	-0.0231	-0.0491**	-0.0551***	-0.0776***	-0.0656*
	(0.0179)	(0.0277)	(0.0240)	(0.0201)	(0.0250)	(0.0346)
control variables	Y	Y	Y	Y	Y	Y
Household economic balance	Ols	Q = 0.10	Q = 0.25	Q = 0.50	Q = 0.75	Q = 0.90
Local entrepreneurship vs. Working out of hometown	0.0050	-0.0065	0.0188	0.0213*	0.0444***	0.0903***
	(0.0513)	(0.0407)	(0.0170)	(0.0122)	(0.0168)	(0.0255)
Nonlocal Entrepreneurship vs. Working out of hometown	-0.1091*	-0.1133**	0.0292	0.0789***	0.1674***	0.3357***
	(0.0578)	(0.0459)	(0.0191)	(0.0138)	(0.0190)	(0.0287)
Working in hometown vs. Working out of hometown	-0.0170	-0.0066	0.0163	0.0003	-0.0132	-0.0260
	(0.0372)	(0.0296)	(0.0123)	(0.0089)	(0.0122)	(0.0185)
control variables	Y	Y	Y	Y	Y	Y

Note: *, **, *** indicate significant at the 10%, 5%, and 1% levels, respectively.

**Table 8:** Marginal effects of employment mode of migrant workers’ household heads on the impact of self-assessed standard of living of different households.

Self-assessed household living standard	Entrepreneurship vs. Work	Local entrepreneurship vs. Working out of hometown	Nonlocal Entrepreneurship vs. Working out of hometown	Working in hometown vs. Working out of hometown
Very low	-0.0105***	-0.0143***	-0.0087***	-0.0035**
Slightly low	-0.0518***	-0.0709***	-0.0430***	-0.0172**
Average	-0.0059***	-0.0079***	-0.0048**	-0.0019*
Slightly high	0.0592***	0.0808***	0.0490***	0.0196**
Very high	0.0090***	0.0123***	0.0074***	0.0030**

Note: *, **, *** indicate significant at the 10%, 5%, and 1% levels, respectively.

Regarding household disposable income, the regression coefficients of local entrepreneurship and foreign entrepreneurship in all magnitudes are significant, and with the increase of magnitudes, the regression coefficients of local entrepreneurship show positive U-shaped fluctuation and the regression coefficients of out-of-hometown entrepreneurship show a rising trend. And the effect of working in hometown on household disposable income passes the significance test at 1% level in both Q = 0.75 and Q = 0.90 quartiles and is negatively correlated.

In terms of per capita consumption, local entrepreneurship and out-of-hometown entrepreneurship have a positive effect on consumption at all quantiles and show positive U-shaped curves with increasing quantiles with Q = 0.25 quantile as the lowest point. Working in hometown has a significant negative effect on consumption at all quantiles and shows a positive U-shaped curve with increasing quantiles with the lowest point at the Q = 0.75 quantile.

However, in terms of household economic balance, for the middle and low population, several employment modes are not much different from each other, and foreign entrepreneurship is instead significant and lowest at Q = 0.10 quantile, and the regression coefficients of local entrepreneurship and foreign entrepreneurship are significant at Q = 0.50, Q = 0.75 and Q = 0.90 quantiles for each of the quantiles, showing an increasing trend.

The results of the marginal effects show that different modes of employment lead to an increase in the self-assessed household standard of living relative to the reference group, but the magnitude is different and is variable. Specifically, the probability of self-assessed household living standard of migrant workers who start their own business compared to those who work in the labor force decreases by 1.05%, the probability of being slightly low decreases by 5.18%, the probability of being average decreases by 0.59%, the probability of being slightly high increases by 5.92%, and the probability of being very high increases by 0.90%. The probability of self-assessed family living standard of migrant workers who started their business locally compared to those who worked abroad decreased by 1.43%, the probability of being slightly low decreased by 7.09%, the probability of being average decreased by 0.79%, the probability of being slightly high increased by 8.08%, and the probability of being very high increased by 1.23%. The probability of self-assessed family living standard of migrant workers who started their business in the field compared to those who worked in the field decreased by 0.87%, the probability of being slightly low decreased by 4.30%, the probability of being average decreased by 0.48%, the probability of being slightly high increased by 4.90%, and the probability of being very high increased by 0.74%. The probability of self-assessed family living standard of migrant workers working locally compared to those working abroad decreased by 0.35%, the probability of being slightly low decreased by 1.72%, the probability of being average decreased by 0.19%, the probability of being slightly high increased by 1.96%, and the probability of being very high increased by 0.30%.

### 4.5. Heterogeneity analysis

In order to better grasp the situation of household effect of employment mode and to understand the variability of the impact of employment mode on household living standard, we grouped the analysis according to the gender and age of household head respectively, and also selected the non-head of household sample in the original database to further analyze the household effect of employment mode of household head and non-head of household, the analysis method and formula are the same as the basic regression, Tables 9 and 10 is obtained.

**Table 9:** Heterogeneity analysis of the effect of employment mode on the living standard of rural migrant workers’ households.

		Household disposable income		Consumption Expenditure per capita		Household economic balance		Self-assessed household living standard	
		Coef.(95% CI)	P	Coef.(95% CI)	P	Coef.(95% CI)	P	Coef.(95% CI)	P
All(n = 5407)									
Gender									
Male									
	Local entrepreneurship vs. doing work out	0.1590(0.0982,0.2197)	0.000	0.0681(0.0182,0.1181)	0.008	0.0222(-0.0770,0.1215)	0.661	0.5556(0.3794,0.7318)	0.000
	Nonlocal entrepreneurship vs. doing work out	0.3937(0.3237,0.4637)	0.000	0.2908(0.2333,0.3484)	0.000	-0.1123(-0.2266,0.0020)	0.054	0.3530(0.1445,0.5616)	0.001
	Doing work locally vs. doing work out	-0.0096(-0.0538,0.0345)	0.669	-0.0527(-0.0890,-0.0164)	0.004	-0.0117(-0.0838,0.0604)	0.751	0.1263(-0.0015,0.2541)	0.053
Female									
	Local entrepreneurship vs. doing work out	-0.3221(-0.5397,-0.1044)	0.004	0.0015(-0.2023,0.2051)	0.989	-0.424(-1.0359,0.1878)	0.174	0.7232(0.02173,1.4247)	0.043
	Nonlocal entrepreneurship vs. doing work out	0.4018(0.2119,0.5918)	0.000	0.4469(0.2691,0.6247)	0.000	-0.1973(-0.7314,0.3368)	0.468	0.2087(-0.404,0.8218)	0.505
	Doing work locally vs. doing work out	-0.3333(-0.4911,-0.1756)	0.000	-0.1207(-0.2683,0.0270)	0.109	-0.2172(-0.6607,0.2262)	0.336	0.2788(-0.2330,0.7907)	0.286
Generation									
New									
	Local entrepreneurship vs. doing work out	0.1645(0.0503,0.2786)	0.005	0.0799(-0.0150,0.1747)	0.099	-0.0454(-0.3057,0.2148)	0.732	0.7519(0.4241,1.0796)	0.000
	Nonlocal entrepreneurship vs. doing work out	0.4308(0.3341,0.5276)	0.000	0.3176(0.2372,0.3980)	0.000	-0.1925(-0.4131,0.0281)	0.087	0.2007(-0.0874,0.4888)	0.172
	Doing work locally vs. doing work out	-0.0386(-0.1258,0.0485)	0.385	-0.0246(-0.0970,0.0479)	0.506	-0.0237(-0.2224,0.1750)	0.815	0.3104(0.0582,0.5627)	0.016
Old									
	Local entrepreneurship vs. doing work out	0.1128(0.0446,0.1810)	0.001	0.0387(-0.0175,0.0948)	0.177	0.0393(-0.0584,0.1370)	0.431	0.4901(0.2901,6902)	0.000
	Nonlocal entrepreneurship vs. doing work out	0.3928(0.3026,0.4829)	0.000	0.3144(0.2402,0.3886)	0.000	-0.0293(-0.1585,0.0998)	0.656	0.4548(0.1844,0.7252)	0.001
	Doing work locally vs. doing work out	-0.0356(-0.0841,0.0129)	0.150	-0.0871(-0.1270,-0.0472)	0.000	0.0094(-0.0600,0.0789)	0.7900	0.0691(-0.0734,0.2116)	0.342

**Table 10:** Impact of different employment modes of non-head of household rural migrant workers on household living standards.

Non-Head of Household	Household disposable income	Consumption Expenditure per capita	Household economic balance	Self-assessed household living standard	Household disposable income	Consumption Expenditure per capita	Household economic balance	Self-assessed household living standard
Entrepreneurship vs. labor	0.2297***	0.1825***	0.0147	0.3556***				
	(0.0245)	(0.0195)	(0.0435)	(0.0646)				
Local entrepreneurship vs. doing work out					0.2569***	0.1983***	-0.0383	0.5925***
					(0.0314)	(0.0256)	(0.0609)	(0.0856)
Nonlocal entrepreneurship vs. doing work out					0.3016***	0.2220***	0.0778	0.2355**
					(0.0373)	(0.0291)	(0.0596)	(0.0943)
Doing work locally vs. doing work out					0.1547***	0.0861***	0.0088	0.2099***
					(0.0202)	(0.0176)	(0.0389)	(0.0609)
Control variables	YES	YES	YES	YES	YES	YES	YES	YES
_cons	5.1539***	7.0752***	10.2833***		5.2856***	7.1484***	10.3008***	
	(0.2522)	(0.2150)	(0.4592)		(0.2516)	(0.2154)	(0.4612)	
R2/	0.120	0.269	0.004		0.125	0.271	0.004	
adj. R2/	0.1185	0.2679	0.0029		0.1240	0.2698	0.0029	
Prob > chi2				0.0000				0.0000
Log pseudolikelihood				-8613.1378				-8602.82
Pseudo R2				0.0192				0.0203
N	8080	8080	8080	8080	8080	8080	8080	8080

Note: Robust standard errors are in parentheses. *, **, *** denote significant at the 10%, 5%, and 1% levels, respectively.

From Tables 9 and 10, it can be seen that.

First, in terms of the gender of the household head, for male household heads, the largest household income effect and the highest level of consumption per capita is generated by foreign entrepreneurship relative to field work, followed by local entrepreneurship. However, at the same time, the balance of income and expenditure of households starting businesses out of town is the lowest. The subjective perception effect shows the advantage of local employment, with the four employment types ranked from highest to lowest being local entrepreneurship, out-of-town entrepreneurship, local work, and out-of-town work. Overall, male-headed household has the best impact on household living standards when they start a business. From the household income effects generated by different employment modes of female household heads, it can be found that the household income effect and level of consumption per capita are the best when female household heads start a business abroad, followed by work abroad, start a business locally, and work in hometown, indicating that female household heads bring a better effect on the household income effect when choosing to start a business out of town, while the subjective perception effect is only relatively more significant for local entrepreneurship than out-of-town work, and there is no significant difference between other employment modes compared to work out of hometown.

Second, in terms of the age of the household head, for household heads born in the 1980s and later, the income effect and the level of per capita consumption are the highest with entrepreneurship outside their hometown compared to working outside their hometown. However, the household balance effect of entrepreneurship outside the hometown is significantly negative, and it ties with out-of-town work for the lowest subjective perception effect. While the income effect and the level of per capita consumption of local entrepreneurship rank second only to out-of-town entrepreneurship among the four modes of employment, the subjective perception effect of local entrepreneurship is the best. Thus, local entrepreneurship is preferable to out-of-hometown entrepreneurship for post-80s heads of household. The new generation of migrant workers should consider living standards at all levels in an integrated manner to make the decision which employment mode to chose. In the case of household heads born prior to the 1980s, the income effect and per capita consumption level derived from entrepreneurship outside their hometown are the most substantial compared to other employment modalities. The subjective perception effect of such entrepreneurship is second only to that of local entrepreneurship. Furthermore, there is no significant decrease in the household’s economic surplus, suggesting that the older cohort of household heads has exerted a positive influence on both the subjective and objective dimensions of family living standards through out-of-town entrepreneurship, without resulting in a deficit.

Third, in terms of whether they are household heads, the effects of non-head employment patterns on household living standards are mostly ranked the same compared to the household head sample, but there are still different findings. The most critical difference lies in the income effect from being hired, the income effect and consumption per capita effect of employed in hometown are better than that of employed out of hometown, while the head of household sample shows no significant difference between the effect of being hired locally on household income and being hired out of hometown, whereas per capita consumption level is notably lower for employed in hometown compared to employed out of hometown.

## 5. Conclusion and discussion

Based on the China Household Income Survey (CHIP), this paper conducted basic regression analysis, heterogeneity analysis and robustness test on the effects brought by the employment mode of household heads on the living standard of rural migrant workers’ households, and came to the following research conclusions: First, the employment mode of household heads has a significant effect on the living standard of rural migrant workers’ households. Specifically, entrepreneurship increases household income by 25.71%, raises per capita consumption expenditure by 19.05%, and enhances the subjective perception of household living standards by 41.27% compared to being employed. Compared with doing work out of hometown, the income effect and per capita consumption expenditure effect of foreign entrepreneurship are the highest of all four employment modes, the subjective perceived effect of local entrepreneurship is highest of all four employment modes, and the subjective perceived effect of local employees is higher than that of nonlocal employees. Secondly, the impacts of different employment methods adopted by migrant worker heads of households with different family living standards vary. Third, using foreign workers as a reference group, when male head of household start a business whether locally or not, the household income effect, per capita consumption expenditure effect and the subjective perception effect behave better; as to female head of household, the income effect and per capita consumption expenditure effect behave better when they are out of hometown no matter start a business or do work for others, but when they chose to start a business locally, their self-assessed household living standard is highest. Fourth, for the pre-80s household heads, starting a business brings better household income effect and per capita consumption expenditure effect than working, and subjective perception effect of local employment are superior to those of out-of-town employment; however, for the post-80s household heads, the impact of local entrepreneurship and being hired out of hometown on per capita consumption levels is roughly equivalent, and the subjective perception effect of starting a business locally is the best, followed by out-of-town entrepreneurship. Fifth, for non-household heads, the household income effects and subjective perception effects from working abroad are significantly lower than those of the other three employment modes, especially the household income effect and per capita consumption effect of working locally are also better than those of working abroad, forming a large difference with the sample of household heads.

Here’s also some issues worth discussing.

**First,** the choice of employment mode and policy guidance for rural migrant workers. The choice of employment mode of rural migrant workers is subjectively influenced by many factors such as human capital and social capital, and objectively influenced by the external environment and national policies, so as to make a choice in line with the law of market supply and demand. From the perspective of family living standard, entrepreneurship provides higher economic returns to rural migrant workers’ families. As for the choice of employment area, it is necessary to take into account various factors, including economic and non-economic factors, and make a decision that is in line with the maximization of their own family welfare. Of course, the choice of employment mode for rural migrant workers should also take into account policy bonus. The "Double Innovation" policy, which encourages "Mass Innovation and Mass Entrepreneurship", was introduced in 2015, and the policy has been continuously improved in terms of promoting entrepreneurship policies, simplifying entrepreneurship procedures and building entrepreneurship incubation bases. Rural migrant workers should actively participate in the process of improving their own labor skills and employment quality to achieve a perfect combination of self-development and social needs.

**Second,** it is about the measurement of the living standard of families and the common prosperity. Living standard is a commonly used term with a rich connotation. From the household perspective, it is the basis of material and spiritual life associated with the level of household income or consumption. From the national point of view, China is now promoting the Common Prosperity of all people with Chinese modernization, which means that all people, including the families of rural migrant workers, should achieve material and spiritual affluence. From this perspective, the living standard of families is one of the important criteria to measure the degree of Common Prosperity, and the living standard of families should not only measure the affluence of their "pockets" but also the affluence of their "heads". Improving the living standard of families is not only a process of accumulating wealth, but also a process of enriching the spirit, which is an important aspect to promote Common Prosperity.

**Third,** the perception of the head of the household and the research value on them. Traditionally, the head of the household is the main pillar and decision maker of the family, and is the spokesman of the family, mostly played by the male. With the reduction of family size and the change of social concept, the traditional status of the head of the household has changed to some extent, but it is still inherited to some extent, especially in rural areas. Among the sample of migrant heads of households studied in this paper, male heads of households should be the majority, accounting for 92.79% and 94.86% married, while female heads of households only account for 7.21% and the proportion of married people drops to 78.21%, but still show the bearing power of women in the family. The research in this paper finds that there are both similarities and differences in the effects of employment modes on household living standards between head of household and non-household heads, male and female household heads, and post-80s and pre-80s household heads. such as why the household income effect of starting a business locally for female household heads is lower than that of working abroad? Why is only the household income effect of working locally better than that of working aboard for non-head of household? What exactly contributes to these and how to understand these differences deserve further research to follow.

This paper focuses on the living standards of rural migrant workers’ households and finds that the employment modes of household heads significantly affect the economic income and perceived living standards of rural migrant workers’ households, while heterogeneity analysis is conducted for household heads of different genders, household heads of different ages, and non-heads of households, yielding some research findings but also leaving some questions worth exploring. The research in this paper confirms that entrepreneurship greatly improves household living standards, but the risk of entrepreneurship is not given enough attention. The biggest problem is that the entrepreneurship in the database is currently existing entrepreneurs, those who have failed or been eliminated are not in the sample, and whether there is a sample bias to expand the entrepreneurship effect is worthy of attention.

## Supporting information

S1 File (DO)

S2 File (DTA)

## References

[1] National Bureau of Statistics of the People’s Republic of China. Migrant Workers Monitoring Survey Report 2021.http://www.stats.gov.cn/tjsj/zxfb/202204/t20220429_1830126.html. 2022.

[2] People’s Daily. A total of 4.5 million migrant workers returned to their hometowns to start businesses.http://www.gov.cn/xinwen/2016-12/02/content_5141650.htm.2016.

[3] Chen Jing. Contribution of Migrant Workers to Economic Growth and the Fruits They Shared Masters Thesis, Jinan University.2013.

[4] Luo M.Z.,& Lei H.K.Non-farm Employment Experience, Psychological Traits and Productivity of New Professional Farmers. Economic and Management Review. 2022(04),124–134. doi: 10.13962/j.cnki.37-1486/f.2022.04.011

[5] Lili Liu. Job Stability and Migrants Workers’Consumption: Theoretical Analysis and Empirical Evidence. Consumer Economics.2021(01),50–58.

[6] Dapeng Sun, Zhiyi Sun, Bintong Yu & Yang Li. Does Non-Agricultural Employment Improve Rural Residents’ Happiness?. Southern Economy.2022(03),17–36. doi: 10.19592/j.cnki.scje.390776

[7] Shengguo Wang & Suzhen Du. Gender Differences in the Effect of One Spouse Starting a Business on Happiness: Based on Empirical Analysis of Social Norms. Economic Management. 2019(12),73–87. doi: 10.19616/j.cnki.bmj.2019.12.005

[8] Qinghua Huang, Ming Zhang, Song Jiang & Xianjin Tu. The effect and mechanism of education on rural residents’ happiness. Agricultural Technology Economics.2017(01),67–75. doi: 10.13246/j.cnki.jae.2017.01.006

[9] Lei Quan, Yuping Chen, Shijun Ding & Haitao Wu. Basic Education, Employment Industry and Family Income of Migrant Workers. Finance and Economics Series.2019(07),3–12. doi: 10.13762/j.cnki.cjlc.20181016.001

[10] Yuanyuan Yue, Congying Wan & Ting Zhao. Study of the Relationship between Entrepreneurship and Happiness of Migrant Workers-Reasearch Based on CFPS Data. Journal of Anhui Normal University Humanities and Social Sciences Edition. 2018 (05), 55–63. doi: 10.14182/j.cnki.j.anu.2018.05.008

[11] Xinxin Mu & Shenghu Chen.Family-Size Effect on Intergenerational Income Mobility under China’s Family Planning Policy: Testing the Quantity-Quality Trade-Off. Sustainability.2022(19),12559–12559. doi: 10.3390/SU141912559

[12] Jun He, Yining Shen & Wenhao Tang. Social Capital, Risk Resistance and Poverty Vulnerability of Rural Female−Headed Households: An Empirical Analysis Based on CFPS. Journal of Nanjing Agricultural University Social Science Edition. 2020(03),146–157.

[13] Yitong Yao, Wang Yapeng& Shen Qingling. Effects of labor migration on farmers’ household income: based on the survey date from four cities in Hubei Province. Zhejiang Journal of Agriculture.2015(04),690–696.

[14] Shiwei Zhang & Na Wu. Income Effects of Training Length on Migrant Workers. Journal of Population.2015 (04), 104–111. doi: 10.16405/j.cnki.1004-129X.2015.04.011

[15] Yanlin Liu. Evaluation of the effect of rural digitalization in improving farmers’ living standards and mechanism research. Guizhou Social Science.2022(02),160–168. doi: 10.13713/j.cnki.cssci.2022.02.006

[16] Yongfu Cao, Mengjie Yang & Yueping Song. Self-employment and income of migrant workers: An empirical analysis based on propensity score. China Rural Economy. 2013(10),30–41+52.

[17] Yusong Liu. Study on the relationship between farmers’ activity of pioneering work and their income based on Chinese data in 1992–2011. Shanghai Journal of Agriculture.2014(05),124–128.

[18] Liqiang Tang & Jing Zhou. Social capital, employment status and rural residents’ non-farm income—an empirical analysis based on CGSS2013 survey data. Rural Economy.2017(05),109–115. CNKI:SUN:NCJJ.0.2017-05-019.

[19] Zhiling Huang. Does Self-employment Increase the Economic Well-being of Peasants’Income?. Agricultural economic issues. 2017(11), 40–47+111. doi: 10.13246/j.cnki.iae.2017.11.005

[20] Zhisheng Zhu. Research on the Rural Migrant worker’s Behavior of Choosing the Self-employment in Urban China Doctoral thesis, Capital University of Economics and Business.2017.

[21] Biyun Yang, Qinbing Mao & Xingjian Yi. Connotation Interpretation, Theoretical Framework and Realization Path of High − Quality Development. Journal of Xiangtan University Philosophy and Social Science Edition.2021(06),32–38+45. doi: 10.13715/j.cnki.jxupss.2021.06.006

[22] Guangjie Ning. Self-selection and regional income differences in nonfarm employment of rural surplus laborers—and whether the Lewis turning point has arrived. Economic Research. 2012(S2),42–55.

[23] Han Junhui& Li Jin. Self-selection, Switch of Non-agricultural Employment between Urban- rural Areas and the Wage Differences. Journal of Yunnan University of Finance and Economics.2015 (04),47–53. doi: 10.16537/j.cnki.jynufe.000023

[24] Zhongjian Li & Lulu Yuan.The Effects of Working Distance on Employment Quality of Rural Migrant Workers. China Rural Economy.2017(06),70–83. CNKI:SUN:ZNJJ.0.2017-06-007.

[25] Yuanrui Hu & Chengzhi Tian. Analysis of the phenomenon of new generation of post-90s migrant workers returning to their hometowns for employment from the perspective of implicit benefits of local migrant workers—Taking Yongning Town of Wenjiang District as an example. China Market. 2016(21),241–243. doi: 10.13939/j.cnki.zgsc.2016.21.241

[26] Xiaopeng Zhen & Chen Ling. The Impact of Rural Labor Migrantion on Rural Income and Income Gap:A labor Heterogeneity Approach. Economics Quarterly.2017(03),1073–1096. doi: 10.13821/j.cnki.ceq.2017.02.11

[27] Xiangfei Yin & Bibo Yin. The Reaearch on Family Head’s Effect on The Urban Household Consumption. Consumer Economics.2017(01),43–49. CNKI:SUN:XFJY.0.2017-01-007.

[28] Jichang Dong, Xiaoting Liu, Ji Kangxian, Dong Zhi & Li Xiuting. Household-head age, housing affordability and tenure choice -Evdence from CFPS. System Engineering Theory and Practice.2021(08),1961–1973. CNKI:SUN:XTLL.0.2021-08-005.

[29] Lu Yajuan& Yin Junyao. Study on the influence of household head’s education on household risky financial asset selection. Journal of Nanjing Audit University. 2021(03),70–80.

[30] Xingjian Yi, Renjing Chen, Lai Te & Yang Biyun. Does the increase in education level of household heads significantly increase the household saving rate J. Shanghai Finance. 2017(11),21–27. doi: 10.13910/j.cnki.shjr.2017.11.003

[31] National Bureau of Statistics of the People’s Republic of China. China Statistical Yearbook 2019. Beijing: China Statistics Press. 2019.

[32] Yujun Lian, Wensu Li, Bihong Huang. The Impact of Children Migration on the Health and Life Satisfaction of Parents Left Behind. Economic Journal, 2014, 14(01): 185–202.

[33] Zhongkun Zhu, Jianping Tao, Chenxin Leng. Migration and Happiness. Southern Economics, 2019, (03): 90–110. doi: 10.19592/j.cnki.scje.350710

[34] Zhichao Yin, Zixuan Zhang. & Yue Pengpeng. Entrepreneurship and household income mobility in China. Research on Quantitative and Technical Economics. 2024(02), 68–88.

